# Costs of treating patients with schizophrenia who have illness-related crisis events

**DOI:** 10.1186/1471-244X-8-72

**Published:** 2008-08-26

**Authors:** Baojin Zhu, Haya Ascher-Svanum, Douglas E Faries, Xiaomei Peng, David Salkever, Eric P Slade

**Affiliations:** 1 Eli Lilly and Company, Indianapolis, USA; 2 University of Maryland, Baltimore County (UMBC), Department of Public Policy, Baltimore, USA; 3 University of Maryland School of Medicine, Baltimore, USA; 4 U.S. Department of Veterans Affairs, VA VISN5 Mental Illness Research and Education Clinical Center, Baltimore, USA

## Abstract

**Background:**

Relatively little is known about the relationship between psychosocial crises and treatment costs for persons with schizophrenia. This naturalistic prospective study assessed the association of recent crises with mental health treatment costs among persons receiving treatment for schizophrenia.

**Methods:**

Data were drawn from a large multi-site, non-interventional study of schizophrenia patients in the United States, conducted between 1997 and 2003. Participants were treated at mental health treatment systems, including the Department of Veterans Affairs (VA) hospitals, community mental health centers, community and state hospitals, and university health care service systems. Total costs over a 1-year period for mental health services and component costs (psychiatric hospitalizations, antipsychotic medications, other psychotropic medications, day treatment, emergency psychiatric services, psychosocial/rehabilitation group therapy, individual therapy, medication management, and case management) were calculated for 1557 patients with complete medical information. Direct mental health treatment costs for patients who had experienced 1 or more of 5 recent crisis events were compared to propensity-matched samples of persons who had not experienced a crisis event. The 5 non-mutually exclusive crisis event subgroups were: suicide attempt in the past 4 weeks (n = 18), psychiatric hospitalization in the past 6 months (n = 240), arrest in the past 6 months (n = 56), violent behaviors in the past 4 weeks (n = 62), and diagnosis of a co-occurring substance use disorder (n = 413).

**Results:**

Across all 5 categories of crisis events, patients who had a recent crisis had higher average annual mental health treatment costs than patients in propensity-score matched comparison samples. Average annual mental health treatment costs were significantly higher for persons who attempted suicide ($46,024), followed by persons with psychiatric hospitalization in the past 6 months ($37,329), persons with prior arrests ($31,081), and persons with violent behaviors ($18,778). Total cost was not significantly higher for those with co-occurring substance use disorder ($19,034).

**Conclusion:**

Recent crises, particularly suicide attempts, psychiatric hospitalizations, and criminal arrests, are predictive of higher mental health treatment costs in schizophrenia patients.

## Background

Schizophrenia is among the most costly of all mental illnesses, with an estimated annual per person direct treatment cost that is approximately 2-fold higher than the cost of major depression and more than 4-fold higher than any anxiety disorder [1]. In addition to genetic factors [2] and medication adherence [3-5], various clinical and demographic factors are associated with higher risk of relapse and hospitalization, events that are predictive of higher costs [6]. These factors include male gender, lower educational level, unemployment, higher illness chronicity, higher frequency of alcohol consumption, co-occurring alcohol and substance abuse, a history of depression and/or suicide attempts, a history of violence and/or arrests, and recent hospitalization [7-10]. The positive association of these factors with mental health treatment costs suggests that persons who are more vulnerable to crises (i.e., personal life events typically associated with an acute societal intervention) have higher treatment costs than persons who are less vulnerable to these events.

The relationship between crisis events and treatment costs has not been well-studied. Most prior studies of costs are based on administrative data that provide relatively little information about vulnerabilities and recent crises [11]. Information about the relationship of crises to costs is essential for accurate risk adjustment, the process of assigning capitation rates for enrollees of public and private health insurance plans. Capitation rates that are set too low provide plans and HMOs insufficient incentive to treat high-risk, high cost patients [12-14]. Information on the effect of crisis events is particularly valuable at a time when there are concerted efforts to decrease patient hospitalization and manage various types of psychiatric crises in the community. In the United States, for example, the length of hospital stay has gradually declined in the past 10 years [15], attesting to economic and policy-driven pressures to reduce psychiatric hospitalizations. Information about crisis events has also clinical utility in usual care settings, where it may help identify more vulnerable patients with more complex illness trajectories who require specialized interventions and better coordination with other social agencies, including the criminal justice system.

Most prospective longitudinal data do not enable the study of whether patients diagnosed with schizophrenia who have experienced a recent crisis incur higher direct mental health costs compared to patients who have not experienced a crisis. However, the availability of comprehensive clinical, functional and economic data from a large 3-year prospective non-interventional observational study of persons treated for schizophrenia in the United States provided the opportunity to address this topic in some detail. The objective of the current investigation was to assess the relationship between recent crisis events and direct annual mental health costs in patients diagnosed with schizophrenia spectrum disorders in clinical practice settings. The crisis events examined included (1) suicide attempt, (2) psychiatric hospitalization, (3) arrest, (4) violent behavior, and (5) substance use disorder. Five separate propensity score analyses were performed (one for each crisis event) to evaluate the relationship between each crisis event and cost when compared to the group of individuals who did not experience that specific crisis event.

## Methods

### Study design

Data used in the current analysis are from the U.S. Schizophrenia Care and Assessment Program (US-SCAP), a prospective, non-interventional, non-randomized, 3-year observational study of more than 2300 persons with schizophrenia. The data used in the current analysis consist of the first full year of data available for each patient in the study. The goal of the US-SCAP was to examine the relationship of clinical and treatment variables with outcomes of persons diagnosed with schizophrenia-spectrum disorders who were receiving mental health care in outpatient and inpatient settings at 6 treatment sites. The study, conducted between July 1997 and September 2003, enrolled a total of 2327 patients at 6 health care sites chosen to provide a diverse patient sample in terms of geography, ethnicity, and clinical setting (e.g., university and community mental health centers, Veterans Affairs (VA) hospitals, and community and state hospitals). Sites were only included in the study if they offered open and unrestricted formulary access to all available antipsychotics and were not relying on any algorithms for treatment decision-making. The study protocol was reviewed and approved by the Institutional Review Board (IRB) at each site prior to study initiation, and written informed consent was obtained from all participants.

A detailed description of study design and methods are provided in previous publications [16-18]. Briefly, US-SCAP study enrollment was offered to all patients who were 18 years or older who had a DSM-IV diagnosis of schizophrenia, schizoaffective, or schizophreniform disorder. Patients were enrolled regardless of psychiatric or medical comorbidity, use of concomitant medications, or presence of behavioral problems (criminal or otherwise). The goal was to obtain the broadest and most representative sample of schizophrenia-spectrum patients seen in clinical practice settings.

For the purposes of the current cost analysis, a subgroup of US-SCAP enrollees (n = 1557; 67%) was identified who had a full year of information available on mental health resource utilization. If patients had >1 year of mental health resource information, the patient's earliest year was used in the current analysis.

Five crisis subgroups were defined based on the presence of any one of the following events at the start of the 1-year observation period: (1) a suicide attempt in 4 weeks prior to the baseline assessment per patient self report; (2) a psychiatric hospitalization in the 6 months prior to baseline per medical records; (3) an arrest in the 6 months prior to baseline per patient self report; (4) violent behavior in the 4 weeks prior to baseline per patient self report; and (5) a diagnosis of substance use disorder, based on medical record, which occurred at any time during the study period.

### Measures

At study entry, patients completed a semi-structured interview, during which information about psychiatric history and background characteristics was collected. Patients' medical records were systematically abstracted every 6 months by examiners, trained and certified by the contract research organization, using a medical record abstraction form developed for this study that summarized mental health resource utilization during the preceding 6 months. Patients were queried about use of medical and psychiatric services outside of their usual treatment site. Study staff members regularly obtained medical records from treatment sites mentioned by patients. At 6-month intervals, patients also completed the SCAP-Health Questionnaire (SCAP-HQ [19]), a personal interview that includes questions on recent drug and alcohol use (in the past 12 months), suicide attempt (in the past 4 weeks), arrests (in the past 6 months), and violent behavior (in the past 4 weeks). At 1-year intervals, clinical assessments of psychiatric symptoms, medication side-effects, and functioning were completed by trained clinicians.

### Assessment of costs

The total annual direct costs were calculated as the sum of the following component charges: medication costs, including antipsychotics and other psychotropics (the cost of antipsychotics was based on Average Wholesale Price discounted by 15% for atypical antipsychotics); costs of psychiatric hospitalization (based on the actual charges); and costs of other mental health services (based on Relative Value Units [RVUs; 20,21] developed from management information systems (MIS) data at each site to help address variations across sites in available information about resource types, costs, and durations). The costs of other mental health services included the following cost components: emergency services, day treatment, outpatient medication management by a physician, individual outpatient therapy, outpatient psychosocial group interventions, and case management.

### Statistical analyses

For comparisons of baseline characteristics, t-tests were used for continuous variables and Mantel-Haenszel chi-square tests for categorical variables. In order to correct for potential bias not attributable to membership in a crisis event subgroup, the propensity score method was used to balance the crisis event versus non-crisis event subgroups. The variables selected *a priori *for calculating the logit score using the propensity method were: age at enrollment, gender, race, illness duration, comorbid affective disorder, comorbid substance use, comorbid personality disorder, diagnosis of mental retardation, insurance status, inpatient status at the beginning of the 1-year observation period, site, and days from July 1 1997 to the beginning of the study. Substance use was not used in the propensity score model when substance use was being analyzed as the crisis event, and inpatient status was not used in the propensity score model when costs associated with recent psychiatric hospitalizations were estimated.

The propensity score-adjusted bootstrapping method analyzed mean differences of costs between each crisis event and non-crisis event group by first calculating the logit score for each patient based on the above adjustments. Five bins of logit scores were then created for each crisis event group. The bootstrap resampling method was performed by randomly selecting an equal size of sample from each of the 10 bins (5 bins for each treatment) into 1 group and calculating the total cost difference between the 2 treatment groups. The above steps were then repeated 1000 times, generating a total of 1000 data points' distribution for testing the null hypothesis using 2-tailed p-values [22]. The propensity score-adjusted bootstrapping method was also used to test the mean differences of costs for the 2 high level crisis event groups: patients with 2 or more crisis-event variables and patients with 3 or more crisis-event variables. This method accommodates the distributional and correlational properties of the data [23]. In addition to mean total cost and cost of psychiatric hospitalization per patient for the index year, we also calculated the mean annual length of psychiatric hospital stay and the mean number of psychiatric hospital admissions per crisis event category. This information aimed at clarifying which type of crisis made a significant and unique contribution to increased costs due to hospitalization, the costliest component in the treatment of schizophrenia. SAS version 8 was used to perform all statistical analyses, with all effects tested at a 2-sided α level of 0.05 [24].

## Results

The total sample used in the cost analysis consisted of the 1557 patients (Table 1) for whom there were at least 1 year of complete medical information during the 3-year study period. For the cost analysis sample, the typical patient was between 30 and 50 years of age, with at least a 10-year history of illness. Almost all (94.9%) of patients were taking an antipsychotic drug at the time of enrollment; 38.3% were also taking an antidepressant, and 31.0% were also taking a mood stabilizer.

**Table 1:** Demographic and clinical characteristics of patients at enrollment

**Characteristic**	**N = 1557**	
	
	**N**	**%**
Male	948	60.9
Single, never married	938	60.5
Ethnicity		
White	762	48.9
Black	589	37.8
Other	206	13.2
Health insurance		
Medicaid/Medicare	1243	81.2
Department of Veteran Affairs	97	6.3
Private insurance	70	4.6
Other coverage	16	1.1
No health insurance	104	6.8
Educational attainment, high school or less	1047	67.9
		
	**Mean**	**SD**
Age, years	42.4	11.1
Age at illness onset, years	20.7	8.9
MADRS total score	13.6	10.2
PANSS total score	69.1	18.4
Medication days in the 6 months prior to enrollment		
Atypical antipsychotic	96.5	87
Typical antipsychotic	90.1	87
Antidepressant	59.1	81.8
Mood stabilizer	46.7	77.3
Other psychotropic	75.5	84.9

PANSS, Positive and Negative Symptoms of Schizophrenia scale

MADRS, Montgomery-Åsberg Depression Rating Scale

Participants who had incomplete medical information for the purpose of costs calculations (N = 770) were excluded from this study. The excluded patients were similar to the analysis sample (N = 1557) in terms of gender (male: 63% vs. 60%; p = .489), but were significantly younger (40.8 ± 11.4 vs. 42.4 ± 11.1 years; p < .01) and were less likely to be African-American (32% vs. 38%), but more likely to be Hispanic (17% vs. 13%) or Caucasian (51% or 49%; overall p = .013). Compared to the 1557 retained subjects, the excluded group was also more likely to have been hospitalized in the prior 6 months (15.4% vs 25.2%, respectively), to be arrested in the prior 6 months (3.8% vs 10.1%), to manifest violent behaviors in the past 4 weeks (4.2% vs 8.4%) and attempt suicide in the past 4 weeks (1.2% vs 3.8%), but had a similar proportion of persons with substance abuse diagnosis (36.1% vs 29.5%). The excluded group may have experienced a greater interface with the criminal justice system (e.g., jails), thus less likely to have complete mental health information in the present study.

**Figure 1:**
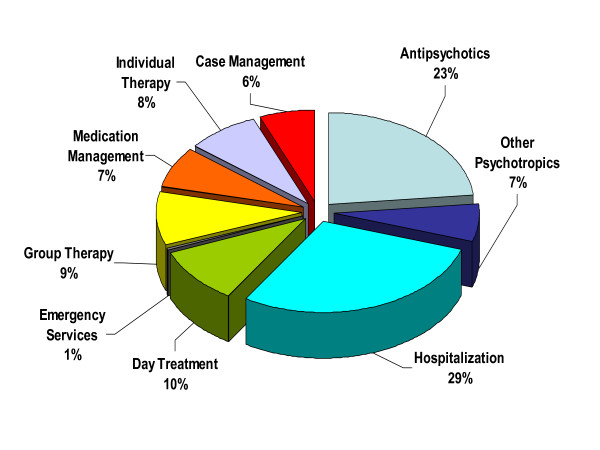
**Mental health cost components as a proportion of total annual mental health costs**. Of the 1-year per patient total mental health treatment cost of $16,098, the largest single contributor was the cost of hospitalization (29%), followed by antipsychotic medication (23%). Each of the remaining 6 cost component categories contributed less than 10% to the total.

The mean 1-year mental health treatment costs per patient totaled $16,098 (Table 2). Te two largest costs were psychotropic medication (30%) and hospitalization (29%; Figure 1). The remaining 6 cost component categories comprised 48% of the total cost, with none contributing more than 10% (Figure 1). Among the hospitalized participants, the correlation between hospitalization costs and total mental health treatment costs during the index year was high (r = .987, p < .001), reflecting the fact that hospitalization is the core driver of total costs.

**Table 2:** Mean 1-year per patient total cost and cost components (n = 1557)

**Cost component**	**Mean Cost ($) ** **(standard deviation)**
Total annual cost	16,098 (24,791)
Medications	4,817 (3,858)
Antipsychotics	3,770 (3,244)
Other psychotropics	1,047 (1,313)
Psychiatric hospitalizations	4,687 (23,536)
Day Treatment	1,571 (3,734)
Emergency Services	84 (196)
Psychosocial group therapy	1,478 (3,126)
Medication management	1,187 (1,331)
Individual therapy	1,267 (1,826)
Case management	1,006 (958)

Sorting patients into crisis event categories yielded 5 overlapping subgroups. The largest subgroups were co-occurring substance use disorder (n = 413) and hospitalized in the past 6 months (n = 240), followed by violent in the past 4 weeks (n = 62), arrested in the past 6 months (n = 56), and attempted suicide in the past 4 weeks (n = 18). Prior to formal matching, each of the crisis event subgroups was not substantially different from each other on most baseline demographic and clinical characteristics (Table 3). The typical patient was most likely to be a male (~61%) between the ages of 30 and 50 years. As expected, the proportion of males was somewhat higher in the subgroup who had been arrested in the past 6 months (80%) and the subgroup with comorbid substance abuse (74%). Though the sample size was small (N = 18), the subgroup who had recently attempted suicide was younger (mean age, 34 years), more likely to be female (61%), and more likely to have a depressive component, with a mean MADRS score of 31, and a diagnosis of schizoaffective disorder (50%; Table 3).

**Table 3:** Characteristics of patients in each crisis event subgroup prior to propensity score matching

	**Crisis events**					
**Characteristic**	**Hospitalized in past 6 months**	**Arrested in past 6 months**	**Violent in past 4 weeks**	**Concurrent substance abuse**	**Attempted suicide in past 4 weeks**	**Non-crisis event subgroup**

Sample sizes						
Yes	240	56	62	413	18	938
No	1317	1469	1463	1144	1507	619
Age, years						
Yes	39 (10.3)	38.9 (9.7)	40.7 (10.0)	39.9 (9.8)	33.8 (11.0)	43.9 (11.4)
No	43 (11.2)	42.6 (11.1)	42.5 (11.1)	43.2 (11.4)	42.6 (11.0)	40 (10.2)
Age of Illness onset, yrs						
Yes	19.8 (8.6)	19.4 (6.3)	18.9 (7.8)	19.4 (8.3)	16.4 (5.8)	21.4 (9.1)
No	20.8 (8.9)	20.8 (9.0)	20.8 (9.0)	21.1 (9.0)	20.8 (9.0)	19.6 (8.5)
MADRS total score						
Yes	15.6 (11.0)	14.0 (10.5)	19.9 (12.0)	13.5 (10.8)	30.9 (12.5)	13.1 (9.8)
No	13.2 (10.0)	13.5 (10.1)	13.3 (10.0)	13.6 (10.0)	13.4 (10.0)	14.3 (10.8)
PANSS total score						
Yes	73.9 (18.4)	71.1 (15.7)	72.0 (18.8)	69.6 (19.1)	86.7 (16.9)	68.0 (17.8)
No	68.2 (18.2)	69.1 (18.5)	69.0 (18.4)	68.9 (18.0)	69.0 (18.3)	70.8 (19.0)
Medication days in 6 mos. prior to enrollment						
Atypicals						
Yes	96.2 (82.9)	61.7 (81.1)	71.2 (84.4)	91.0 (87.0)	89.3 (82.2)	98.6 (87.5)
No	96.6 (87.7)	97.7 (86.9)	97.4 (86.9)	98.5 (86.9)	96.5 (87.0)	93.4 (86.2)
Typicals						
Yes	72.2 (82.0)	95.9 (82.9)	98.4 (88.8)	98.1 (86.5)	60.9 (80.8)	90.3 (87.6)
No	93. 4 (87.4)	89.8 (87.1)	89.7 (86.9)	87.2 (86.9)	90.4 (87.0)	89.7 (85.8)
Antidepressant						
Yes	54.2 (78.9)	55.1 (81.0)	71.2 (84.4)	53.1 (80.0)	74.7 (86.6)	61.3 (82.6)
No	60(82.3)	59.6 (82.0)	58.9 (81.8)	61.3 (82.4)	59.2 (81.9)	55.9 (80.6)
Mood stabilizer						
Yes	64.1 (82.0)	41.3 (74.3)	46.4 (77.1)	38.7 (71.7)	61.0 (84.1)	45.5 (77.3)
No	43.5 (76.0)	47.0 (77.5)	46.8 (77.4)	49.6 (79.1)	46.6 (77.3)	48.6 (77.4)
Other psychotropic						
Yes	71.0 (80.5)	63.8 (79.9)	99.1 (82.5)	79.3 (85.4)	36.1 (63.3)	74.1 (85.5)
No	76.4 (85.7)	75.7 (85.0)	74.3 (84.8)	74.2 (84.8)	75.7 (85.0)	77.7 (84.2)
Male, N (%)						
Yes	142 (59.2)	45 (80.4)	38 (61.3)	304 (73.6)	7 (38.9)	527 (56.2)
No	806 (61.2)	882 (60.0)	889 (60.8)	644 (56.3)	920 (61.0)	421 (68.0)
Single, N (%)						
Yes	149 (62.3)	46 (82.0)	26 (41.9)	262 (63.8)	11 (61.1)	549 (58.8)
No	789 (60.2)	871 (59.6)	891 (61.2)	676 (59.4)	906 (60.4)	389 (63.2)
Diagnosis, N (%)						
Schizophrenia						
Yes	119 (49.6)	33 (58.9)	32 (51.6)	257 (62.2)	9 (50.0)	620 (66.1)
No	861 (65.4)	926 (63.0)	927 (63.4)	723 (63.2)	950 (63.0)	360 (58.2)
Schizoaffective						
Yes	103 (42.9)	17 (30.4)	28 (45.2)	125 (30.3)	9 (50.0)	282 (30.1)
No	393 (29.8)	469 (31.9)	458 (31.3)	371 (32.4)	477 (31.7)	214 (34.6)
Other psychotic						
Yes	19 (7.9)	6 (10.7)	2 (3.2)	31 (7.5)	0	38 (4.1)
No	65 (4.9)	77 (5.2)	81 (5.5)	53 (4.6)	83 (5.5)	46 (7.4)

Means (standard deviations) unless otherwise specified. Patients may belong to more than one subgroup. Yes = with event; No = without event.

PANSS, Positive and Negative Symptoms of Schizophrenia scale

MADRS, Montgomery-Åsberg Depression Rating Scale

Atypicals = atypical antipsychotics

Typicals = typical antipsychotics

The five crisis event categories were not mutually exclusive (Table 4), with highest levels of overlap between substance use disorder, arrest, suicide attempt and violent behavior. A substantial proportion (46.4%) of those with arrest, with suicide attempt (44.4%), and with violent behaviors (41.9%) had also a diagnosis of substance abuse. While 21.1% of those with substance abuse diagnosis were hospitalized, about a third (36.3%) of the hospitalized had co-occurring diagnosis of substance abuse. Furthermore, participants with substance use disorders had the lowest mean annual number of hospitalization days (25.5) and a relatively low mean annual number of psychiatric admissions (0.7).

**Table 4:** Proportion of participants in each crisis event category and degree of overlap between categories

	**Hospitalized in prior 6 months**	**Arrested in previous 6 months**	**Violent behavior in previous 4 weeks**	**Concurrent substance abuse diagnosis**	**Attempted suicide in past 4 weeks**
**Hospitalized in prior 6 months ** **(N = 240)**	__	22 (9.2%)	17 (7.1%)	87 (36.3%)	11 (4.6%)
**Arrested in previous 6 months ** **(N = 56)**	22 (39.3%)	__	5 (8.9%)	26 (46.4%)	1 (1.8%)
**Violent behavior in previous 4 weeks ** **(N = 62)**	17 (27.4%)	5 (8.1%)	__	26 (41.9%)	2 (3.2%)
**Concurrent substance abuse diagnosis ** **(N = 413)**	87 (21.1%)	26 (6.6%)	26 (6.6%)	__	8 (2.0%)
**Attempted suicide in past 4 weeks ** **(N = 18)**	11 (61.1%)	1 (5.6%)	2 (11.1%)	8 (44.4%)	__

The 1-year hospitalization and total 1-year mental health treatment costs were calculated for each crisis event subgroup, and compared, using a propensity score adjusted bootstrap re-sampling method (repeated 1000 times), to the non-crisis event subgroup. The results of this analysis (summarized in Table 5) found significantly higher mean 1-year hospitalization costs, and total mental health costs, for each crisis event subgroup except comorbid substance abuse. The mean total annual mental health cost for the subgroup who had experienced no crisis event was $11,739 per patient. The mean total mental health cost for patients with at least 1 crisis event was $22,704 per patient, with the highest cost observed in the subgroup of patients who attempted suicide, followed by patients with a recent psychiatric hospitalization, arrest, violent behavior, and those with comorbid substance use disorder (Table 5). Of the patients (n = 619) who qualified for inclusion in at least 1 crisis event subgroup, 30.9% had experienced only one crisis event, 8.9% had experienced 2 or more crisis events, and 1.9% had experienced 3 or more events. The presence of ≥2 and ≥3 crisis events was associated with a significant and step-wise increase in both 1-year hospitalization and total 1-year mental health costs compared to the costs in the non-crisis event subgroup, reflecting a high mean number of hospitalization days (72.7 and 101.1 days, respectively) and a high mean number of hospital admissions (1.7 and 2.7, respectively) (Table 6).

**Table 5:** Mean 1-year total costs, hospitalization costs and hospitalization parameters for patients with and without specific crisis event categories

	**Patients with event**		**Mean 1-year total mental health cost per patient**	**Mean 1-year cost of psych hospitalization per patient**	**Mean number of days hospitalized**	**Mean number of hospital admissions**
	**N**	**%**				
Hospitalization in previous 6 months						
Yes	240	15.4	$37,329 **	$23,962 **	73.3	1.9
No	1317	84.6	$12,229	$1,175	4.8	0.3
Arrested in previous 6 months						
Yes	56	3.7	$31,081 *	$20,334 *	55.8	1.1
No	1469	96.3	$15,654	$4,180	14.1	0.5
Violent behavior in previous 4 weeks						
Yes	62	4.1	$18,778 **	$7,416 **	29.6	1.3
No	1463	95.9	$16,113	$4,661	15.1	0.5
Concurrent substance abuse disorder						
Yes	413	26.5	$19,034	$7,455	25.5	0.7
No	1144	73.5	$15,038	$3,688	11.7	0.5
Attempted suicide in past 4 weeks						
Yes	18	1.2	$46,024 **	$30,080 **	61.8	1.3
No	1507	98.8	$15,865	$4,471	15.1	0.5

* P < .05; ** P < .01; P values compare non-crisis event group cost ("no") vs. crisis event group cost ("yes") for each event.

**Table 6:** Mean 1-year costs for patients with and without specific number of crisis event categories

	**Number of types of crisis events**			
	**None**	**One only**	**Two or more**	**Three or more**

Patients with event, N (%)	938 (60.2%)	481 (30.9%)	138 (8.9%)	29 (1.9%)
Mean 1-year cost of hospitalization per patient	$830	$6,912 *	$23,149 *	$33,199 *
Mean 1-year total mental health cost per patient	$11,739	$19,066 *	$35,385 *	$44,599 *
Mean number of days hospitalized	3.7	21.8	72.7	101.1
Mean number of hospital admissions	0.2	0.8	1.7	2.7

* P < .01; P values based on non-crisis event group vs. each category: patients with only one type of crisis event, two or more types of events, and three of more types of crisis events.

## Discussion

Previous research has identified various clinical variables as significant predictors of relapse and hospitalization in patients diagnosed with schizophrenia. The current study extends these findings by providing, for the first time, information on the annual mental health costs associated with experiencing specific crisis events. The use of comprehensive assessments in a prospective naturalistic study with valid and reliable instruments enabled the identification of individuals who had experienced specific crisis events, and the systematic collection of resource utilization data for different types of mental health services. Given the large and diverse sample of schizophrenia patients analyzed and the prospective, naturalistic design, the findings of the study are likely to be applicable to patients with schizophrenia treated in large systems of care across the United States.

Although 60.2% of the participants did not report experiencing a crisis event, 39.8% of the patients did, with 30.9% of all participants meeting only one crisis event criterion, 8.9% meeting 2 or more criteria, and about 2% meeting three or more criteria. Thus, the base rate for experiencing a crisis event was not low.

As hypothesized, patients experiencing a crisis event that took place (or at least started) before the 1-year observation period accounted for a preponderance of the total mental health costs during that period. Furthermore, a disproportionate contribution to increased mental health costs was made by the subgroup who reported experiencing 2 or more crisis events. The per patient mean total annual mental health cost for the non-crisis event subgroup was $11,739, while patients who reported at least 1 crisis event had a per patient mean of $22,704. (Note that the mean total costs shown in Table 5 for each crisis event subgroup are higher, because some of the individuals in each group may have experienced 1 or more additional crisis events.)

The highest annual per patient total mental health cost was in the subgroup of patients who attempted suicide, followed by patients with a recent psychiatric hospitalization. In the current investigation, patients with a concurrent substance use disorder did not appear to have significantly higher total mental health costs compared to those without a substance use disorder. This is an unexpected finding since substance abuse has previously been reported to be a predictor of medication nonadherence [25,26], which, in turn, is often reported to be a highly significant predictor of future relapse and hospitalization [27-29]. One possible explanation is suggested by recent data from the Clinical Antipsychotic Trials in Intervention Effectiveness (CATIE) study which found that substance abuse was a marker for higher psychosocial functioning in schizophrenia [30]. Another possibility is that some of the substance abusing participants – especially those with prior arrests and violent behaviors- had more extensive interface with the criminal justice system (e.g., jails), thus some of their costs may have shifted from the mental health to the criminal justice system. In addition, nonadherence with medication may also underlie the higher costs incurred by patients in the other crisis event categories, since nonadherence with antipsychotic medication was previously found to be associated not only with psychiatric hospitalizations but also with a higher risk of violent behaviors, arrests and suicide attempts [29]. While detecting ongoing medication nonadherence may be difficult and challenging in usual care settings, the occurrence of a crisis event is apt to be more readily identifiable, thus serve as a clinical marker of patients' greater vulnerability from a clinical and functional perspective. This may turn out to be a convenient way of targeting subsets of patients with different illness profiles (e.g., with different diagnostic characteristics, illness trajectories, or vulnerability factors) and effectively treating individuals who experience crisis events.

Cost differences among patients who experience a crisis event appeared to be primarily driven by the cost of psychiatric hospitalization, with a strong and significant correlation (r = .99) between hospitalization cost and total annual mental health costs. In the current analysis, hospitalization comprised, however, only 29% of the total annual mental health costs, while antipsychotic medication comprised 23%. This finding is inconsistent with prior research in which the cost attributed to hospitalization was of larger proportion- about 50%–80% – of the total cost [31,32]. The reason for the inconsistency is unclear. The relatively low percentage attributed to hospitalization may reflect the way that costs were calculated in the present study. Specifically, the cost of psychiatric hospitalization was based on actual charges while the cost of antipsychotics was based on Average Wholesale Price discounted by 15% and the cost of other non-hospitalization mental health services was based on Relative Value Units. It is possible, therefore, that the methods of estimating non-hospitalization costs led to their overestimation relative to hospitalization cost. Furthermore, the relative cost contribution of hospitalization and antipsychotic medication to overall total direct costs would be substantially lower in the current study if non-psychiatric medical costs and direct non-health care costs had been available for analysis.

### Study limitations

The study has important limitations which deserve to be highlighted. First, the criteria for several of the crisis event subgroups (attempted suicide, arrested, violent) were based on a patient-reported measure, not on objective data. It should also be noted that substance abuse is not, strictly speaking, a discrete event, but an ongoing condition. Second, the sample sizes for several of the crisis event subgroups were small, most notably for the "attempted suicide" group. The appropriateness of the propensity score adjusted bootstrap re-sampling method with such small sample sizes may be questionable. Third, some hospitalizations may have been underreported since stays in state psychiatric hospitals may not have been reported by all patients, and such hospitalizations would have been missed by most of the MIS systems. Fourth, emergency department visits may have also be significantly underreported, since they would not have been captured by MIS or by medical record in most cases. This might help to explain why the estimate of emergency department costs was negligible in the current study. Fifth, an estimation bias may have occurred in the calculation of medication costs using average wholesale price (AWP; discounted by 15% for atypical antipsychotics). The AWP may not reflect variations in medication costs and greater discount rates across different health care systems. A final limitation is that the estimates reported in this paper are for mental health costs only and do not include non-psychiatric medical costs or direct non-health care costs (e.g., patient involvement in the criminal justice system, use of homeless shelters) – the latter alone have been estimated at $9.3 billion per year [2]. The omission of these cost categories has likely resulted, in the current analysis, in an overestimate of the relative contribution of some cost categories. Future cost studies should include data on direct non-health care costs, especially since the mental health cost burden is being increasingly shifted to the criminal justice system.

## Conclusion

Mental health costs of treating patients diagnosed with schizophrenia and related disorders are highly heterogeneous. Patients who experience crisis events, particularly those with a recent suicide attempt or psychiatric hospitalization, tend to incur the highest annual mental health costs, driven primarily by the cost of psychiatric hospitalization. Patients involved in the criminal justice system (with prior arrest) also accrue relatively high costs within the mental health delivery system. More prospective research, in usual care settings, is needed to identify high risk patients and to determine which interventions are the most cost effective.

## Abbreviations

AWP: average wholesale price; CATIE: Clinical Antipsychotic Trials in Intervention Effectiveness; MADRS: Montgomery-Åsburg Depression Rating Scale; MIS: management information system; PANSS: Positive and Negative Syndrome Scale; RVUs: Relative Value Units; SCAP-HQ: SCAP-Health Questionnaire; TD: tardive dyskinesia; US-SCAP: U.S. Schizophrenia Care and Assessment Program; VA: Veterans Affairs.

## Competing interests

Baojin Zhu, Haya Ascher-Svanum, Douglas Faries and Xiaomei Peng are full-time employees and minor stockholders of Eli Lilly and Company, the sponsor of the study.

David Salkever and Eric P. Slade have no competing financial or non-financial interests.

## Authors' contributions

BZ performed the statistical analyses, participated in the design of the study, the analytical plan, the interpretation of the results, and assisted in drafting the manuscript. HA–S conceived of the study, participated in its design, the analytical plan, the interpretation of the results, and helped draft the manuscript. DEF participated in the design of the study, the analytical plan, the interpretation of the results, and assisted in drafting the manuscript. XP performed additional statistical analyses. DS and EPS participated in the design of the study, the analytical plan, the interpretation of the results, and assisted in drafting the manuscript. DS and EPS were also involved in preparing the resource utilization costing data of US-SCAP.

## Pre-publication history

The pre-publication history for this paper can be accessed here:


